# One Hundredth Anniversary of the University of Maryland

**Published:** 1907-07

**Authors:** 


### ONE HUNDREDTH ANNIVERSARY OF THE UNIVERSITY OF MARYLAND.

A notable event in the history of medical education in the United States was the recent centennial of the University of Maryland. About 6,000 men have graduated from this school and many of them have taken first rank in promoting the scientific and the general professional advancement of medicine. In a country comparatively young, one hundred years is a long time for continued service, and this college is indeed to be congratulated upon its career. Many distinguished men have taught in its various chairs and have been the inspiration of many worthy practitioners as well as men of fame whose names are inseparably connected with pioneer work in many branches of medicine.

St. John's College of Annapolis, has just been amalgamated with the University of Maryland and comes as a partial consummation of a plan proposed during the administration of Gov. Paca in 1784, and now makes of it a university in fact as in name. Keen appreciation was shown the greetings and expressions of felicity which were sent from most of the well-known institutions of learning in the world. The review of its century of achievement on an occasion marked by so much enthusiasm and impressiveness will undoubtedly stimulate the faculty and students of this school to greater efforts and will instill new life and determination in the alumni to strive to be worthy of so honored an Alma Mater.

